# The Etiological and Antimicrobial Susceptibility Profiles of the Bacteria Obtained from Ovine Caseous Lymphadenitis Cases in the Çankırı Region, Türkiye

**DOI:** 10.3390/life14091078

**Published:** 2024-08-28

**Authors:** Serdal Tarhane, Fatih Büyük

**Affiliations:** 1 Veterinary Department, Eldivan Vocational School of Health Services, Çankırı Karatekin University, Çankırı 18200, Türkiye; 2 Microbiology Department, Faculty of Veterinary Medicine, Kafkas University, Kars 36300, Türkiye; fatihbyk08@hotmail.com

**Keywords:** 16S RNA sequence, AST, CLA, culture, phylogeny, sheep

## Abstract

Sheep caseous lymphadenitis (CLA) causes significant economic losses in the livestock sector by causing a loss in the quantity and quality of animal products and a loss in the breeding value of animals. Although the primary agent in CLA’s etiology is *Corynebacterium pseudotuberculosis*, some other opportunistic microorganisms also play a role. Therefore, the control and treatment of CLA necessitates the identification of the relevant etiological agents. This study aimed to conduct an in vitro culture and molecular characterization (PCR analysis and 16S rRNA sequencing) of the bacteria involved in sheep CLA cases reported in the Çankırı province of Türkiye and determine the antibiotic susceptibility of the case isolates. In total, 82 (16.4%) of 500 sheep in five farms were diagnosed with CLA. Following the culture of the superficial abscesses samples, *C. pseudotuberculosis* was identified in 30 (36.59%) as a result of PCR, *Pseudomonas* spp. in 8 (9.76%), and *Enterobacter cancerogenus* in 1 (1.22%), as a result of 16S rRNA sequencing. These data revealed extensive heterogeneity among the *Pseudomonas* isolates, with hints of derivation from a common ancestry for some and phylogenetic similarity to isolates from Germany, Malaysia, and India. In contrast to the high susceptibility to cefoperazone and lincomycin, the high resistances of *C. pseudotuberculosis* and *Pseudomonas* spp. isolates to cephalothin, ceftiofur, cloxacillin, amoxicillin, and bacitracin were remarkable. Based on these findings, it was concluded that for an effective treatment and control of ovine CLA cases, there is a need to consider the possible involvement of opportunistic bacteria other than the primary causative agent, *C. pseudotuberculosis*. It also contributed to increasing the country-specific sequence data and establishing new taxa from a universal perspective.

## 1. Introduction

Animal breeding has an important place in global agricultural production and a share of 25% of all agricultural activities in Türkiye. Sheep breeding has an important place in small ruminant production, and the global sheep population was estimated as 1.1 billion heads in 2007. Türkiye ranks ninth in the world for its sheep population of 25,400,000 heads [1,2,3].

Ovine and caprine cases of caseous lymphadenitis (CLA) cause major economic losses due to adverse effects on meat, skin, and wool production. Furthermore, ovine and caprine CLA are also known to cause carcass losses, the loss of breeder animals, and a reduced circulation of animal products, due to infected animals being slaughtered [4]. CLA is described as a disease that is hard to control, due to the etiological agents frequently not responding to treatment and the disease being difficult to detect in the event of a subclinical course [5].

CLA is of bacterial origin and is described as a very common, chronic, and sporadic disease of sheep and goats. The primary causative agent of the disease is *Corynebacterium pseudotuberculosis*, which is a Gram-positive, asporogenic, and facultative anaerobic bacterium [6]. Today, CLA is still highly prevalent in sheep and goats. Recent research has shown that ovine CLA cases are not only caused by *C. pseudotuberculosis* but also by other bacteria, including among others, *Staphylococcus aureus* subsp. *anaerobius*, *Pseudomonas aeruginosa*, *Pseudomonas hydrophila*, *Treuperella pyogenes*, *Staphylococcus lentus*, and *Actinomyces hyovaginalis* [6,7].

Today, CLA is observed in sheep flocks all around the world. The mean prevalence of CLA in adult sheep in West Australia was reported as 58% in 1973 and 53% in 1984. In a slaughterhouse study conducted in Australia, meat inspection demonstrated the presence of signs of infection in 54% of the adult sheep carcasses and 3.4% of the lamb carcasses [8]. In another slaughterhouse study, the prevalence of CLA was observed to rise up to 61% in the adult animals of individual flocks raised in Tasmania and West Australia [9]. Subsequent studies demonstrated a continuous decrease in the prevalence of CLA, which was attributed to the introduction of a prophylactic vaccine in 1983 that gained popularity in the farmer community. In a field study carried out on 412 sheep flocks in West Australia, the mean prevalence of the disease was reported as 45% [10]. Reports have indicated the sensitivity of *C. pseudotuberculosis* to several antibiotics under in vitro conditions. However, this in vitro antimicrobial activity generally proves to be ineffective in clinical practice due to the thick capsule and dense pus content of abscesses that form in CLA cases. Nonetheless, following the resection of the lesion by surgical intervention, parenteral antibiotic treatment is administered [11,12].

The control of CLA, which is characterized by multi-etiology, requires the identification of the causative agents and the determination of their phenotypic and genotypic behavior. In this context, the present study was aimed at the investigation of the in vitro culturable bacteria, isolated from caseous lymphadenitis cases diagnosed in sheep flocks raised in the Çankırı region of Türkiye, using conventional (bacterial culture and phenotypic tests) and molecular (PCR and gene sequence analyses) methods, and the determination of the antimicrobial sensitivity of the case isolates.

## 2. Material and Method

### 2.1. Acquisition of Samples

The study material comprised samples of abscess content from sheep diagnosed with caseous lymphadenitis in flocks raised in the Çankırı province and its vicinity in 2023. The samples were taken with the aid of sterile syringes from abscesses localized to the neck region of 82 animals, from 5 different flocks composed of a total of 500 sheep. The samples were stored at −80 °C until being used (maximum two weeks) for bacterial culture and molecular analyses.

All analyses of the biological samples at risk of contamination were carried out in BSL2 Plus laboratories equipped with the biosafety cabinets belonging to the Department of Microbiology, Faculty of Veterinary Medicine, Kafkas University.

### 2.2. Bacterial Culture

The abscess samples were inoculated onto 5% defibrinated sheep blood agar and incubated in an aerobic environment at 37 °C for 48 h. The bacteria, which formed the colonies that grew on the media, were assessed for their Gram-staining characteristics and microscopic morphology. The initial identification of *Corynebacterium* spp. was performed using phenotypic methods (colony morphology, hemolytic activity, and microscopic morphology) [13,14]. The other culture-positive isolates were identified by 16S rRNA gene sequence analysis [15].

### 2.3. C. Pseudotuberculosis-Specific PCR

DNA extraction from pure bacterial colonies was performed using a commercial kit in accordance with the manufacturer’s instructions (DNeasy Blood & Tissue Kit^®^, Hilden, Germany). For the identification of *C. pseudotuberculosis* by PCR, primers PLDF, PLDR1, and PLDR2, targeting the *pld* gene, were used. The positive control strain was obtained from the Microbiology Department of Kafkas University, Faculty of Veterinary Medicine. DNA/RNA-free water was used as the negative control. The primers, PCR reaction mixture, and PCR thermal cycle are shown in Table 1. The PCR products were run on a 1.5% agarose gel, imaged with an UV trans-illuminator, and documented, and the 203 bp-bands were considered to be *C. pseudotuberculosis* [13].

### 2.4. 16S rRNA Gene Sequencing and Phylogenetic Analysis

DNA extraction from pure bacterial colonies was performed using a commercial kit in accordance with the manufacturer’s instructions (DNeasy Blood & Tissue Kit^®^, Hilden, Germany). The Gram-negative bacteria were subjected to 16S rRNA-PCR and then 16S rRNA gene sequence analysis. For this purpose, the 27F and 1492R primers were used in the 16S rRNA-PCR amplification and sequencing. The primers, PCR reaction mixture, and PCR thermal cycle are shown in Table 1. The PCR products were run on a 1.5% agarose gel by electrophoresis, and the 1465 bp-products of the 16S rRNA gene were assessed. The PCR products were sequenced using the 27F and 1492R primers. For phylogenetic analysis, the resulting chromatograms were compared to the reference sequences available in the BLAST (Basic Local Alignment Search Tool) database of the National Center for Biotechnology Information (NCBI). The base identification of the chromatograms was performed by visual inspection using Finch TV (version V1.4). The evolutional analyses were performed using MEGA 11 software [16].

### 2.5. Antibiotic Sensitivity Test

The antibiotic sensitivity tests of the identified bacterial isolates were performed with the Kirby–Bauer disc diffusion test. For this purpose, firstly, fresh cultures of the bacteria were prepared on the appropriate solid growth media. Bacterial inocula adjusted to McFarland 0.5 were prepared with sterile physiological saline. Volumes of 100 µL of the bacterial inocula were streaked onto Mueller Hinton agar with a sterile loop. After the inoculum was absorbed, antibiotic discs containing 30 µg of cephalothin (KF), 30 µg of ceftiofur (EFT), 5 µg of cloxacillin (OBS), 10 µg of streptomycin (S), 75 µg of cefoperazone (CFP), 25 µg of amoxicillin (AML), 109 µg of lincomycin (LS), 40 µg of penicillin (PNV), and 10 units of bacitracin (BCDD) were placed onto the agar. The bacterial isolates inoculated onto Mueller Hinton agar plates containing these antibiotic discs were incubated at 37 °C for 24 h. The diameters of the inhibition zones were measured and assessed according to the guidelines of the Clinical and Laboratory Standards Institute [17].

## 3. Results

**Figure 1:**
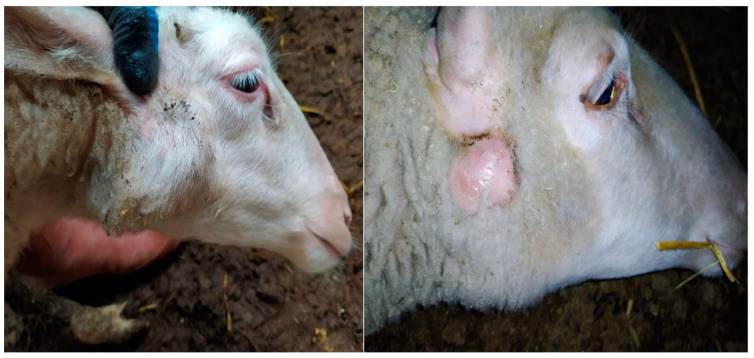
The sampled abscesses located in the neck region of the sheep.

In this study, from five flocks composed of a total of 500 sheep, 82 animals (16.4%) with superficial neck abscesses were sampled for abscess content (Figure 1). It was determined that all of the sampled animals were female and breeder ewes.

### 3.1. Bacterial Culture Findings

**Figure 2:**
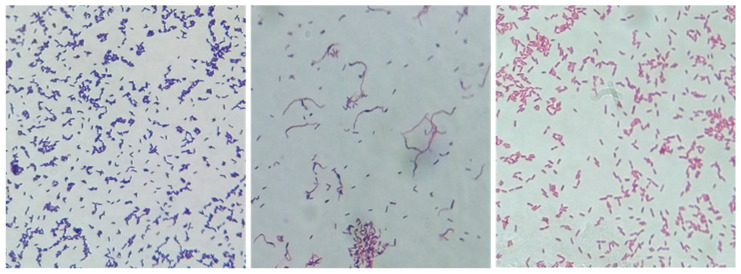
*C. pseudotuberculosis* and the microscopic appearance of the mixed culture and other Gram-negative isolates (magnification 1000×).

Out of the 82 samples, 39 (47.56%) were culture-positive. Of the derived isolates, 28 were Gram-positive bacilli (rods), 7 were Gram-negative bacilli and coccobacilli, and 2 displayed a mixed morphology (Figure 2). After the mixed isolates were purified, two were determined to be Gram-positive bacilli. The other two isolates were determined to be Gram-negative bacilli. Thus, 30 isolates were considered as *Corynebacterium* spp., based on their initial phenotypical characteristics.

### 3.2. C. Pseudotuberculosis-Specific PCR Findings

**Figure 3:**
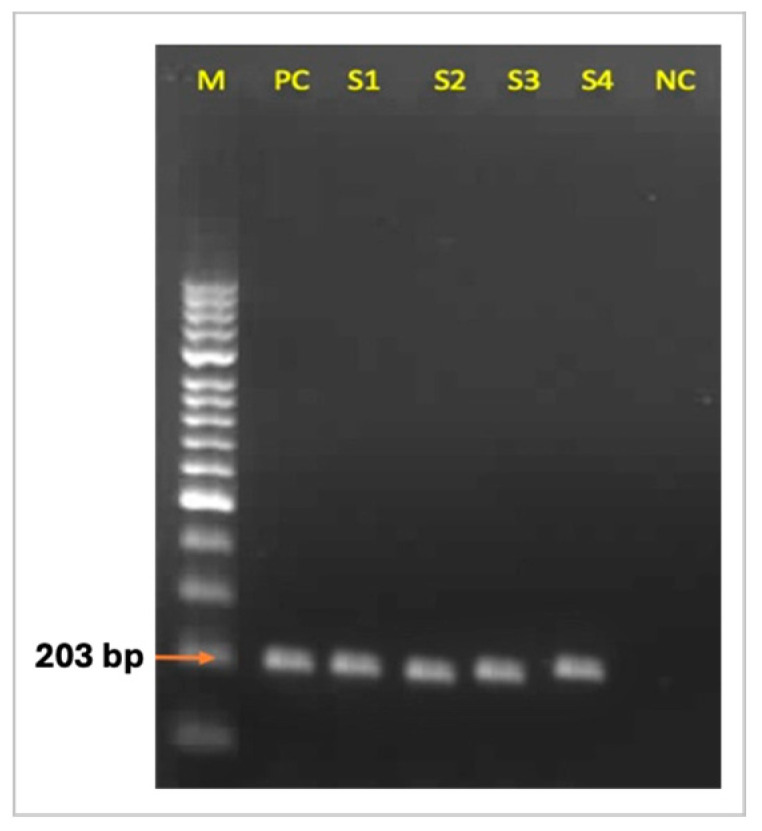
The electrophoresis of the PCR products specific to the *pld* gene. M: DNA ladder (Gene ruler 100 bp plus DNA ladder SM0321, Thermo Fischer Sci., Waltham, MA, USA). PC: positive control. S1, S2, S2, S3, S4: *C. pseudotuberculosis* field isolates. NC: negative control.

The species-specific PCR analysis yielded a 203 bp-band for each of the 30 isolates identified as *Corynebacterium* spp. by phenotypical tests, and thus, all of these isolates were confirmed to be *C. pseudotuberculosis* (Figure 3).

### 3.3. 16S rRNA Gene Sequencing and Phylogenetic Analysis Findings

As a result of the sequence analysis of the 16S rRNA-PCR products with 1465 bp, the Gram-negative isolates were identified as 8 *Pseudomonas* spp. and 1 *Enterobacter cancerogenus* (OR512110). The *Pseudomonas* isolates were identified as *Pseudomonas helleri* (OR512107), *Pseudomonas synxantha* (OR512109), *Pseudomonas moraviensis* (OR512111), *Pseudomonas libanensis* (OR512104), *Pseudomonas fluorescens* (OR512105), *Pseudomonas fluorescens* (OR512106), *Pseudomonas azotoformans* (OR512112), and *Pseudomonas gessardii* (OR512108) (Table 2).

**Figure 4:**
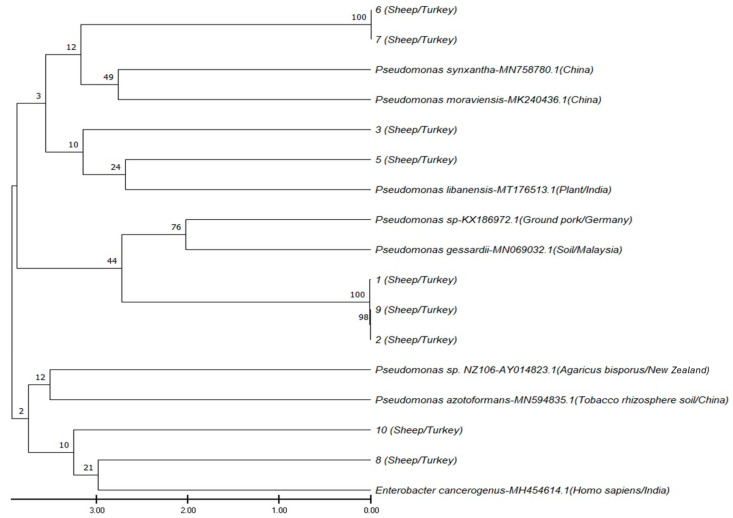
Neighbor-joining phylogenetic tree constructed with 16S rRNA gene sequences showing the phylogenetic position of the isolates among the related taxa. Bar, 0.005 substitutions per nucleotide position.

The isolates obtained in the present study showed a highly heterogeneous distribution under two main clusters. The first cluster of the dendrogram included the isolates numbered 1, 2, 3, 5, 6, 7, and 9, which exhibited a paraphyletic distribution. Isolates 6 and 7 showed 100% similarity to each other, and likewise, isolates 1, 2, and 9 also showed 100% similarity among themselves and, thus, were characterized by a monophyletic distribution. Isolates 6 and 7 and isolates 1, 2, and 9 belonged to distant yet identical branches. The remaining isolates of the first cluster (isolates 3 and 5) presented with a paraphyletic distribution. The second cluster of the dendrogram included isolates 8 and 10, which also displayed a paraphyletic distribution (Figure 4).

Isolates 6 and 7, which were considered to have descended from a common ancestor, were included in the first cluster of the dendrogram. The isolates of this cluster, which showed a high level of similarity to each other (100%), were determined to belong to different taxa, based on comparison with GenBank sequences. Isolates 6 and 7 were determined to belong to taxa close to *Pseudomonas synxantha* strain JM20 (MN758780.1) and *Pseudomonas moraviensis* strain WTB8 (MK240436.1). The other three isolates of the first cluster, which showed 100% similarity to each other, namely isolates 1, 9, and 2, were determined to belong to a taxon close to *Pseudomonas* spp. strain TH21 (KX186972.1), which was isolated from minced pork in Germany in 2012, and *Pseudomonas gessardii* strain ST3SE (MN069032.1), isolated from soil in Malaysia. The paraphyletically distributed isolate 5 of the first cluster belonged to the same taxon with *Pseudomonas libanensis* strain DST45 (MT176513.1), which was isolated from plants in India in 2018. Isolate 3 belonged to a taxon close to that of isolate 5. As for the isolates included in the second cluster of the dendrogram, isolate 8 belonged to the same taxon as *E. cancerogenus* strain MB30 (MH454614.1), which was isolated from humans in India in 2018, and isolate 10 belonged to a taxon close to that of isolate 8 (Figure 4).

### 3.4. Antibiotic Sensitivity Test Findings

While 27 (90%) of the *C. pseudotuberculosis* isolates were found to be sensitive to cefoperazone and lincomycin, 3 (10%) were found to be resistant to these antibiotics. Furthermore, 18 (60%) of the *C. pseudotuberculosis* isolates showed sensitivity, and 12 (40%) showed resistance to penicillin. All (100%) of the *C. pseudotuberculosis* isolates were determined to be resistant to cephalothin, ceftiofur, cloxacillin, streptomycin, amoxicillin, and bacitracin (Table 3).

Out of the eight *Pseudomanas* spp. isolates, all (100%) were sensitive to cefoperazone, seven (87.5%) were sensitive to lincomycin, one (12.5%) was resistant to lincomycin, one (12.5%) was sensitive to streptomycin, and seven (87.5%) were resistant to streptomycin. *Pseudomonas* spp. were found to be resistant to all of the other tested antibiotics (cephalothin, ceftiofur, cloxacillin, amoxicillin, penicillin, and bacitracin) (Table 4).

## 4. Discussion

Caseous lymphadenitis (CLA), caused by *C. pseudotuberculosis*, still occurs and bears importance in regions, where sheep are raised [11]. The prevalence of CLA varies among countries. At the beginning of the nineties, an unavoidable incidence of CLA occurred in sheep and goat flocks raised in the United Kingdom, which caused great loss to the livestock sector [11,18]. While the primary causative agent involved in the etiology of CLA is known to be *C. pseudotuberculosis*, several reports have also indicated the involvement of secondary microorganisms. In a study carried out in Iraq, PCR results revealed *C. pseudotuberculosis* positivity for 22 (2.1%) out of 1090 abscesses detected in sheep and goat carcasses at a slaughterhouse [19]. In Egypt [20], researchers screened 1450 animals by molecular and cultural methods, detected CLA in 24 sheep, and reported the prevalence of *C. pseudotuberculosis* as 1.65%. In the same study, 10 of the ovine CLA cases were determined to have been caused by *C. pseudotuberculosis* alone, whilst in the other CLA cases, *Staphylococcus aureus* and *Streptococcus pyogenes* were isolated and identified together with *C. pseudotuberculosis* by bacterial culture. In a study on the prevalence of CLA in Kosovo, *C. pseudotuberculosis* was identified from 38 (84.2%) out of 284 ovine abscess samples that underwent cultural analysis and biochemical testing. In this study, the prevalence of CLA was determined as 11.3%, and the most prevalent agents after *C. pseudotuberculosis* were determined as *Staphylococcus aureus* (4/38, 10.5%) and *Streptococcus pyogenes* (2/38, 5.3%) [21]. In the present study, a total of 500 sheep were screened, and 82 were determined to have mandibular abscesses and were found to be positive for CLA. Our findings, indicating an overall CLA prevalence of 16.4%, are similar to those reported by Mazreku et al. [21] but differ from those reported by Ali et al. [20] and point out to a CLA positivity higher than those reported by these researchers. The results of the present study support the proposition of Baird and Fountain [11] that CLA prevalence varies among countries and regions and continues to be a major health problem for sheep and goats.

Previous studies carried out in sheep and goats have demonstrated that the causative agents of ovine and caprine CLA are not limited to *C. pseudotuberculasis* and *S. aureus* subsp. *anaerobius* and may include several other bacteria, namely *P. aeruginosa*, *P. hydrophila*, *Treuperella pyogenes*, *Staphylococcus lentus*, *Actinomyces hyovaginalis*, *Streptococcus ovis*, *Staphylococcus simulans*, *Staphylococcus equorum*, *Staphylococcus caprae*, and *Staphylococcus warneri* [7,22]. In a study conducted by Tiwari et al. [23] in India, the bacteria isolated and identified from 255 skin samples of various animal species, including cattle, buffaloes, dogs, goats, sheep, camels, and horses, among others, were *Staphylococcus aureus* (36.22%), *E. coli* (34.59%), *Pseudomonas* spp. (20.54%), *Bacillus* spp. (16.21%), *Klebsiella* spp. (12.43%), *Micrococcus* spp. (8.11%), *Streptococcus pyogenes* (7.56%), *Proteus* spp. (6.49%), *Clostridium* spp. (3.78%), a Gram-positive asporogenic rod (2.16%), and *Fusobacterium* spp. (0.54%). In another study conducted in Saudi Arabia, it was reported that bacteria were isolated from 97 (80.83%) out of 120 ovine abscess samples, and that the remaining 23 samples were sterile. In the same study, identification by the API-20 method revealed that the bacteria in 52 of the samples were *C. pseudotuberculosis* and *S. aureus* subsp. *anaerobius* and that these species constituted 54% of the isolates. The bacteria in the remaining 45 samples were identified as pyogenic bacteria, such as *S. aureus*, *Streptococcus spp.*, *Pseudomonas aeruginosa*, and *Actinomyces pyogenes*, and these were ascertained to constitute approximately 46% of all the isolates. Based on these results, the low level of protection attained with the use of CLA vaccines was attributed to the abscesses having been caused by different bacterial species [5].

Although CLA has been shown to be contracted by the intranasal, intravenous, and intravaginal routes, the primary route of infection has been reported as the contamination of skin wounds. Such wounds may result from fights among animals, grooming, ear-tagging or the brushing of skin against dirty surfaces [24,25]. *P. aeruginosa* is an opportunistic environmental pathogen commonly found in soil, water, and barn flooring and may readily enter the body through skin lesions, eventually resulting in skin, ear, mammary gland, and lung infections in sheep [26]. Although known to be less virulent than *P. aeruginosa*, other *Pseudomonas* species, including *P. fluorescens*, *P. luteola*, *P. putida*, and *P. stutzeri* have been reported to be involved in various infections in immunocompromised hosts [27]. On the other hand, *Enterobacter cancerogenus* is a facultative aerobic bacterium belonging to the family *Enterobacreiaceae* and is commonly found in water, sewage, soil, and plants. Described as being an opportunistic pathogen, *E. cancerogenus* has been reported to may cause pneumonia, bacteremia, post-neurosurgical meningitis, skin and soft tissue infections, and urinary infections in both humans and animals [28,29]. In the present study, while most of the ovine CLA cases (36.59%) were determined to have been caused by *C. pseudotuberculosis*, the bacteria identified from 9.52% and 1.19% of the cases were *Pseudomonas* spp. and *E. cancerogenus*, respectively. This supports the opinions of Umer et al. [25] and Latif et al. [24] that factors, which disrupt the skin barrier, and thereby cause predisposition to microbial contamination and enable infection with opportunistic bacteria other than the primary causative agent of CLA, *C. pseudotuberculosis*, such as *Pseudomanas* spp. and *E. cancerogenus*. In fact, there are literature reports pointing out the isolation of these opportunistic pathogens from cases of CLA [5,7].

It has been reported that the administration of antibacterial protocols for the treatment of animals with caseous lymphadenitis may not result in acceptable levels of antimicrobial efficiency. Due to the abscesses being surrounded by a thick capsule and the causative microorganisms showing intracellular localization, it is indicated that some antibiotics may not reach effective concentrations within the abscesses [30]. In a study conducted in Egypt, 38 *C. pseudotuberculosis* isolates from 80 sheep and 36 goats were determined to be highly resistant to penicillin (96.2%) and erythromycin (92.3%) but highly sensitive to ciprofloxacin (96.2%), amikacin (90.4%), and neomycin (88.5%) [31]. Furthermore, out of 59 *C. pseudotuberculosis* isolates from 614 goats and 154 sheep destined for slaughter in Ethiopia, 35 (59.32%) were found to be sensitive to ampicillin and clindamycin, 43 (72.88%) were determined to be sensitive to doxycycline and tetracycline (72.88%), 41 (69.49%) were ascertained to be sensitive to vancomycin and kanamycin, and 46 (77.97%) were identified as being sensitive to norfloxacin [32]. Antibiotic sensitivity tests performed by El Damaty et al. [33] on 54 (24.5%) *C. pseudotuberculosis* isolates from CLA cases diagnosed in 150 sheep and 90 goats in the Sharkia state of Egypt demonstrated that, while all isolates (100%) were resistant to bacitracin and florphenicol, all isolates (100%) were sensitive to norfloxacin. The same study showed that while most of the isolates were highly resistant to penicillin and erythromycin (92.6% for each) as well as to cefradine (88.9%), 7.4% of the *C. pseudotuberculosis* isolates were resistant to vancomycin. Furthermore, out of 16 *C. pseudotuberculosis* isolates derived from 67 sheep raised in the Balıkesir province of Türkiye, sensitivity was determined in 14 (87.5%) to enrofloxacin, 13 (81.2%) to neomycin, bacitracin, and tetracycline, 12 (75.0%) to penicillin and novobiocin, 11 (68.7%) to oxytetracycline, 11 (68.7%) to amoxicillin and clavulanic acid, 10 (62.5%) to cloxacillin, 9 (56.2%) to tetracycline, 5 (31.2%) to ampicillin and sulbactam, and 3 (18.7%) to trimethoprim and sulfamethoxazole. In the present study, *C. pseudotuberculosis* isolates were tested for sensitivity to ten different antibiotics, such that 27 (90%) were found to be sensitive to cefoperazone and lincomycin, and 18 (60%) were determined to be sensitive to penicillin. All of the isolates were found to be resistant to cephalothin, ceftiofur, cloxacillin, streptomycin, amoxicillin, and bacitracin. The differences in the findings obtained in the present study and in previous research was attributed to the different study regions and the different antibiotics used for treatment, as suggested by Ilhan [34].

16S rRNA gene sequencing is described as a rather powerful tool for the identification of bacteria isolated from various sources [15]. While the use of physiological and biochemical characteristics has been proven to be of use for the classification of the genus *Pseudomonas*, it has been reported to fail in differentiating between *Pseudomonas* species. Nonetheless, 16S rRNA gene sequencing has also been reported to be an effective method for the identification of *Pseudomonas*-like microorganisms [35]. In the present study, excluding *C. pseudotuberculosis*, all of the other bacterial isolates were identified at the species level by means of 16S rRNA gene sequencing.

16S rRNA gene sequencing is frequently used in epidemiological and phylogenetic analyses [15]. It can reveal possible common ancestor epidemics and strain similarities from different geographies due to the border between countries. However, since there is no uniformity for the division of *Pseudomonas* isolates into various clusters after 16S rRNA sequencing, bacterial isolates obtained from different locations generally do not fully overlap each other and are take place in different taxa. In the present study, in addition to the widespread heterogeneity among the *Pseudomonas* isolates and the clues that some isolates came from a common ancestor (6 and 7 or 1, 2, and 9), it was determined that the isolates were phylogenetically related to isolates from Germany, Malaysia, and India, which are geographically distant from the country under study. The sequence data of the isolates obtained from CLA in our country are quite limited, and until the increase in sequence data and the creation and reinterpretation of new taxa, it is open to discussion that they are in the same taxon with isolates from countries that are not geographically close to this study’s country, unless there is a possible international animal movement.

The genus *Pseudomonas* includes more than 200 species, which are Gram-negative bacteria commonly found in animals, soil, and plants [36]. *Pseudomonas* spp. belong to the subclass of Gammaproteobacteria and show various life cycles in a wide environmental array from soil, water, and plant surfaces to animals. These bacteria are known for their prevalence in nature, the capability of using various organic compounds as energy sources, resistance to many medicinal and agricultural antimicrobial substances, and the ability to generate high levels of secondary metabolites [37]. Among all antibiotic-resistant pathogens, *P. aeruginosa* is especially highlighted for its continuously increasing resistance to antimicrobial agents, resulting in treatment complications and failures [38]. Several literature reports have been published on the occurrence of multiple antibiotic resistance in *P. aeruginosa* isolates from ovine CLA cases and other clinical cases of infection [39,40,41]; yet, there are only limited data available on the AST profiles of other *Pseudomonas* species and environmental isolates [42]. In a study carried out in Egypt on a total of 260 sheep and goats with respiratory infection, diarrhea, and abscessed skin lesions, 13 isolates were identified as *P. aeruginosa* by biochemical tests, and each of these isolates was found to be resistant to ampicillin, bacitracin, erythromycin, streptomycin, tetracycline, trimethoprim–sulfamethoxazole, and tobramycin and sensitive to ceftriaxone and norfloxacin. While all of the nine *Pseudomonas* species (100%) identified in the present study were found to be sensitive to cefoperazone, eight (89%) were determined to be sensitive to lincomycin, and one (11%) was ascertained to be sensitive to streptomycin. On the other hand, all of the *Pseudomonas* species identified in the present study were found to be resistant to cephalothin, ceftiofur, cloxacillin, cefoperazone, penicillin, and bacitracin. These results are attributed to the development of multiple antibiotic resistance in *Pseudomonas* species due to the nonselective use of antibiotics, as suggested by Dapgh et al. [39].

## 5. Conclusions

In conclusion, similar to the case in other countries, ovine CLA remains important in terms of animal health in Türkiye. Although the primary causative agent of CLA is known to be *C. pseudotuberculosis*, the possible involvement of other opportunistic bacteria (*Pseudomonas* spp., *E. concerogenus*) in the etiology of CLA should not be overlooked. We highlight the importance of considering the potential involvement of these secondary agents in CLA for achieving success in protection from the disease, as well as in the control and treatment of CLA. Furthermore, phylogenetic clustering of the strains can contribute to increasing country-specific sequence data and creating new taxa from a universal perspective.

## Figures and Tables

**Table 1:** Species-specific and 16S rRNA-PCR conditions of the isolates.

Species	Primer Sequence (5′—3′)	Target Gene	PCR Component (25 μL)	Thermal Cycle	Amplicon (bp)	Reference
*C. pseudotuberculosis*	PLDF- ATAAGCGTAAGCAGGGAGCA PLDR1- ATCAGCGGTGATTGTCTTCC PLDR2- ATCAGCGGTGATTGTCTTCCAGG	*pld*	2.5 μL PCR buffer (×10) 2.5 μL MgCl_2_ (25 mM) 0.5 dNTP (10 mM) 1 μL each primer (10 pmol) 0.5 μL Taq DNA polymerase (5 U) 2.5 μL template DNA (40–80 ng/μL) 13.5/14.5 μL nuclease free water	One cycle: Denaturation at 94 °C for 5 min Thirty-five cycles: Denaturation at 94 °C for 1 min Annealing at 56 °C for 1 min Elongation at 72 °C for 2 min One cycle: Final elongation at 72 °C for 5 min	203	[13]
Unknown species specific	27F- AGAGTTTGATC(AC)TGGCTCAG 1492R- ACGG(CT)TTACCTTGTTACGACTT	*16S rRNA*		One cycle: Denaturation at 95 °C for 5 min Thirty cycles: Denaturation at 94 °C for 15 s Annealing at 59 °C for 30 s Elongation at 72 °C for 45 s One cycle: Final elongation at 72 °C for 5 min	1465	[16]

**Table 2:** The Gram-negative bacteria identified by 16S rRNA gene sequencing.

Number of Isolate	Cultural and Phenotypic Characteristics	Species Name	NCBI Accession Number
1	Gram-negative, bacilli	*P. helleri*	OR512107
2	Gram-negative, bacilli	*P. synxantha*	OR512109
3	Gram-negative, bacilli	*P. moraviensis*	OR512111
5	Gram-negative, bacilli	*P. libanensis*	OR512104
6	Gram-negative, bacilli	*P. fluorescens*	OR512105
7	Gram-negative, bacilli	*P. fluorescens*	OR512106
8	Gram-negative, bacilli	*P. azotoformans*	OR512112
9	Gram-negative, bacilli	*P. gessardii*	OR512108
10	Gram-negative, cocobacilli	*E. cancerogenus*	OR512110

**Table 3:** Antibiotic sensitivity test results of *C. pseudotuberculosis*.

Antibiotic (Concentration)	Susceptible	Intermediate	Resistant
KF (30 µg)	0 (0%)	0 (0%)	30 (100%)
EFT (30 µg)	0 (0%)	0 (0%)	30 (100%)
OBS (5 µg)	0 (0%)	0 (0%)	30 (100%)
CFP (75 µg)	27 (90%)	0 (0%)	3 (10%)
AML (25 µg)	0 (0%)	0 (0%)	30 (100%)
LS (109 µg)	27 (90%)	0 (0%)	3 (10%)
PNV (40 µg)	18 (60%)	0 (0%)	12 (40%)
S (10 U)	0 (0%)	0 (0%)	30 (100%)
BCDD (10 U)	0 (0%)	0 (0%)	30 (100%)

KF: cephalothin, EFT: ceftiofur, OBS: cloxacillin, S: streptomycin, CFP: cefoperazone, AML: amoxicillin, LS: lincomycin, PNV: penicillin, BCDD: bacitracin.

**Table 4:** Antibiotic sensitivity test results of *Pseudomonas* spp.

Antibiotic (Concentration)	Susceptible	Intermediate	Resistant
KF (30 µg)	0 (0%)	0 (0%)	8 (100%)
EFT (30 µg)	0 (100%)	0 (0%)	8 (100%)
OBS (5 µg)	0 (0%)	0 (0%)	8 (100%)
CFP (75 µg)	8 (100%)	0 (0%)	0 (0%)
AML (25 µg)	0 (100%)	1 (11%)	8 (89%)
LS (109 µg)	7 (87.5%)	0 (0%)	1 (12.5%)
PNV (40 µg)	0 (0%)	0 (0%)	8 (0%)
S (10 U)	1 (12.5%)	4 (50%)	3 (37.5%)
BCDD (10 U)	0 (0%)	0 (0%)	8 (100%)

KF: cephalothin, EFT: ceftiofur, OBS: cloxacillin, S: streptomycin, CFP: cefoperazone, AML: amoxicillin, LS: lincomycin, PNV: penicillin, BCDD: bacitracin.

## Data Availability

Data are contained within the article.
